# Supplementary material for: Symptomatic Chikungunya Virus Infection and Pregnancy Outcomes: A Nested Case-Control Study in French Guiana
Source: Viruses. 2022 Dec 2;14(12):2705. doi: 10.3390/v14122705 (PMC9787529; doi:10.3390/v14122705)
Supplement: Supplementary file 1 [file viruses-14-02705-s001.zip › viruses-2026815-supplementary.pdf]

Table S1: Description and measure of outcomes in the pregCHIK study;

Outcomes	Definitions and measures
Pregnancy outcomes	
Preterm delivery	Delivery before 37 weeks of gestation (WG) and after 22 WG of a live newborn weighing more than 500 grams, excluding miscarriages.
Preterm labor	A modification of the cervix associated with regular uterine contractions (ultrasound and fetal monitoring)
preeclampsia	Gestational hypertension with proteinuria (>=300mg/24h) after 20 WG.
Stillbirth	The birth of a dead infant who weighed >500 grams or at or after 22 WG.
Fetal growth restriction	Rate of fetal growth that is below normal in light of the growth potential of a specific infant.
Postpartum hemorrhage	Loss of more than 500 ml of blood during delivery or immediately post-partum (within 5 days of delivery).
Neonatal outcomes	
Apgar score	The five signs of the Apgar score are heart rate, respiratory effort, muscle tone, reflex irritability, and color.
Respiratory distress	Respiratory distress of the newborn defined as the need for cardiopulmonary assistance at birth or in the immediate postpartum period
Congenital anomalies	structural or functional abnormalities that occur during intrauterine life and are diagnosed in the immediate postpartum period
NICU admission	transfer of the newborn at birth or in the immediate postpartum period to an intensive care unit (ICU or Kangaroo Unit)
Macrosomia	Birth weight > 4,000 grams
Small for gestational age infants	Birth weight below the 10th percentile for gestational age according Intergrowth-21st classification
